# Flexible Epoxy Resin Formed Upon Blending with a Triblock Copolymer through Reaction-Induced Microphase Separation

**DOI:** 10.3390/ma9060449

**Published:** 2016-06-03

**Authors:** Wei-Cheng Chu, Wei-Sheng Lin, Shiao-Wei Kuo

**Affiliations:** Center for Nanoscience and Nanotechnology, Department of Materials and Optoelectronic Science, National Sun Yat-Sen University, Kaohsiung 804, Taiwan; m993100044@student.nsysu.edu.tw (W.-C.C.); jjll6213@gmail.com (W.-S.L.)

**Keywords:** epoxy, reaction-induced microphase separation, F127, block copolymer

## Abstract

In this study, we used diglycidyl ether bisphenol A (DGEBA) as a matrix, the ABA block copolymer poly(ethylene oxide–*b*–propylene oxide–*b*–ethylene oxide) (Pluronic F127) as an additive, and diphenyl diaminosulfone (DDS) as a curing agent to prepare flexible epoxy resins through reaction-induced microphase separation (RIMPS). Fourier transform infrared spectroscopy confirmed the existence of hydrogen bonding between the poly(ethylene oxide) segment of F127 and the OH groups of the DGEBA resin. Small-angle X-ray scattering, atomic force microscopy, and transmission electron microscopy all revealed evidence for the microphase separation of F127 within the epoxy resin. Glass transition temperature (*T*_g_) phenomena and mechanical properties (modulus) were determined through differential scanning calorimetry and dynamic mechanical analysis, respectively, of samples at various blend compositions. The modulus data provided evidence for the formation of wormlike micelle structures, through a RIMPS mechanism, in the flexible epoxy resin upon blending with the F127 triblock copolymer.

## 1. Introduction

Epoxy resins have good mechanical integrity, high thermal stability, ready processability, good chemical resistance to solvents and moisture, and exceptional adhesion to many materials. In industry, the versatility in their formulations makes epoxy resins convenient to use and widely applicable—for example, in surface coatings, painting materials, adhesives, composites, insulating materials for electrical devices, and encapsulates for semiconductors [1,2]. One disadvantage of common epoxy resins, however, is that they can be extremely brittle. Accordingly, many attempts have been made to improve the toughness of epoxy materials, generally in two different ways. The first approach involves the addition of an agent with soft segments (e.g., siloxane moieties) into the main or modifying side chains of the epoxy resins [3]; this approach can be quite complex, require expensive initial materials, and be limited to laboratory settings when exploiting new epoxy resin materials. The second method involves blending with nanofillers [4] or polymers (e.g., polystyrene [5], liquid rubber [6], clay and polysiloxanes [7]) as toughness agents; these modified epoxies with mechanical flexibility can be prepared on large scales, using simple approaches, with cheap initial materials [8,9,10,11,12,13].

In recent years, many attempts have been made to enhance the toughness of crisp epoxy resins through the self-assembly of amphiphilic block copolymers that form ordered or disordered nanostructures within thermoset polymers [14,15,16,17,18]. These amphiphilic block copolymers often contained block segments that are miscible and immiscible with epoxy resin; for example, the poly(ethylene oxide) (PEO) block segment is a miscible block segment that has been used most widely to obtain self-assembled nanostructures within epoxy resins [18]. Intermolecular hydrogen bonding interactions with the epoxy resin are often exploited to improve the miscibility. Similarly, such amphiphilic self-assembling block copolymers have also been added into a range of other thermoset resins, including polybenzoxazine resins and phenolic resins [19,20,21,22,23,24,25,26,27,28,29,30,31,32,33].

**Figure 1:**
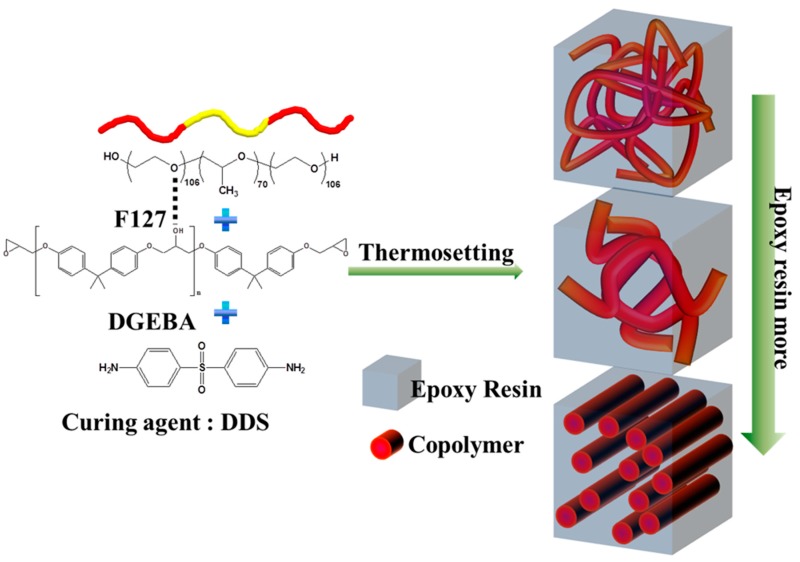
Schematic representation of epoxy resins blended with the triblock copolymer.

In this study, we prepared flexible thermosetting epoxy resins through blending with the ABA triblock copolymer poly(ethylene oxide–*b*–propylene oxide–*b*–ethylene oxide) (EO_106_PO_70_EO_106_, Pluronic F127). We expected that the OH groups of diglycidyl ether bisphenol A (DGEBA) would hydrogen bond primarily with the ether groups of the PEO segment of F127 at relatively low DGEBA concentrations, but would hydrogen bond with the ether groups of both the PEO and poly(propylene oxide) (PPO) blocks at relatively higher DGEBA resin concentrations, most likely forming a series of composition-dependent nanostructures [34,35]. Figure 1 displays possible structures for epoxy resins templated with the triblock copolymer F127. Herein, we used Fourier transform infrared (FTIR) spectroscopy to investigate the hydrogen bonding interactions within these flexible epoxy resins; small-angle X-ray scattering (SAXS), transmission electron microscopy (TEM), and atomic force microscopy (AFM) to examine their microphase separation behavior; and differential scanning calorimetry (DSC) and dynamic mechanical analysis (DMA) to study their mechanical and thermal properties.

## 2. Experimental

### 2.1. Materials

Pluronic F127 (EO_106_PO_70_EO_106_; *M*_n_ = 12,600), diphenyl diaminosulfone (DDS), and tetrahydrofuran (THF) were purchased from Aldrich. DGEBA (DER 331, epoxy equivalent weight = 190 g/equiv) was purchased from Nan-Ya Chemical of Taiwan (Kaohsiung, Taiwan).

### 2.2. Synthesis of Flexible Epoxy Resins

The DGEBA epoxy resin, DDS (curing agent), and pluronic F127 (flexibility agent) were dissolved in THF to form a homogenous solution. The solvent was evaporated slowly at room temperature, and then the samples were dried at 40 °C overnight under vacuum. The samples were subsequently cured using the following temperature profile: 100 °C for 3 h, 150 °C for 3 h, and 200 °C for 0.5 h (heating rate: 2 °C/min).

### 2.3. Characterization

A Q-20 differential scanning calorimeter (TA Instruments, New Castle, CA, USA) was used to perform the thermal analysis. The DSC instrument was operated at heating and cooling rates of 20 and 5 °C/min, respectively, at temperatures from +250 to −80 °C under N_2_, with samples weighing between 5 and 10 mg. FTIR spectra were recorded using a Bruker Tensor 27 spectrophotometer (Billerica, MA, USA); samples were prepared using the common KBr disk method. SAXS was performed using a Bruker Nanostar U small-angle X-ray scattering system (Billerica, MA, USA) with Cu Kα radiation (40 kV, 35 mA). A Leica Ultracut UCT microtome (Wetzlar, Germany) equipped with a glass knife was used to prepare ultrathin sections of the TEM samples (thickness: *ca*. 100 nm). TEM experiments were performed using a JEOL 2100 microscope (Tokyo, Japan) operated at 200 kV. AFM experiments were performed using an environmentally controlled AFM5300E atomic force microscope (Hitachi, Tokyo, Japan), allowing observations under various conditions. DMA was performed using a Q800 apparatus (TA Instruments, DuPont, New Castle, CA, USA) operated in a single-cantilever bending mode at temperatures ranging from −90 to +200 °C. Data acquisition was performed automatically by the system for analysis of the storage modulus (*E*_0_) and loss tangent (tan *δ*). The heating rate was fixed at 2 °C/min and frequency was maintained at 1 Hz, respectively. Samples for the DMA experiments were prepared through molding with dimensions of 30 mm × 8 mm × 2 mm.

## 3. Results and Discussion

### 3.1. FTIR Spectroscopic Analyses of DGEBA + DDS/F127 Block Copolymer Blends

**Figure 2:**
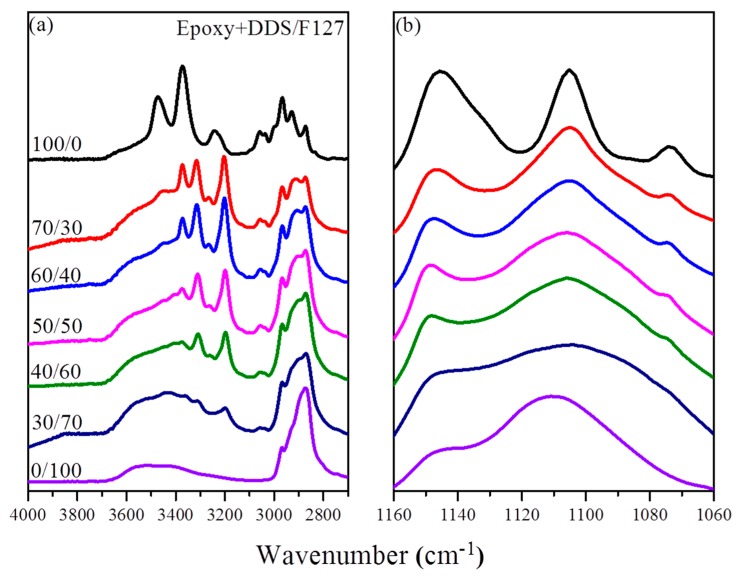
Room-temperature Fourier transform infrared (FTIR) spectra of epoxy/F127 blends, displaying the (**a**) OH stretching and (**b**) ether regions.

We prepared blends of the DGEBA resin, the curing agent DDS, and the common triblock copolymer F127 as the template at various weight ratios (Table 1). FTIR spectroscopy is a commonly used method for examining hydrogen bonding interactions in polymer blend systems. Here, we used FTIR spectroscopy to ascertain whether hydrogen bonding was occurring within the (DGEBA + DDS)/F127 blend system. In Figure 2a, the signals in the room-temperature FTIR spectra of the DGEBA/F127 blend in the region of OH stretching were simple, suggesting the existence of hydrogen bonds. The spectrum of pure DGEBA featured a characteristic major unresolved broad peak at 3480 cm^−1^, corresponding to the signal of its free OH groups; this peak’s intensity decreased.

Gradually upon increasing the concentration of the triblock copolymer F127. Sharp peaks centered at 3373 and 3245 cm^−1^ appeared for DDS, representing the self-association hydrogen bonding interactions of its NH_2_ groups. The broad peak centered at 3480 cm^–1^ for DGEBA became broader when it was blended with F127 at increasing DGEBA concentrations, suggesting that hydrogen bonding was occurring between the OH groups of DGEBA and the ether groups of both the PEO and PPO segments. At lower DGEBA concentrations, the signal for the OH groups involved in [OH**···**ether] hydrogen bonds with the PEO segment appeared at 3275 cm^−1^ [36], suggesting that the interaction strength of the hydrogen bonds followed the order [OH**···**ether] > [OH**···**OH].

Figure 2b displays the signals for C–O–C stretching of the PEO and PPO block segments after blending with (DGEBA + DDS). In a previous report, the free ether groups of PEO and PPO segments were observed near 1112 and 1104 cm^−1^, respectively; furthermore, the signals of the ether groups of the PEO and PPO block segments hydrogen bonded to the OH groups of DGEBA were noted at 1090 and 1082 cm^−1^, respectively [36]. In addition, the signal for the ether units of the triblock copolymer F127 was located at 1109 cm^−1^, representing the free ether groups of the PEO and PPO block segments. This peak shifted to relatively higher wavenumber upon decreasing the DGEBA concentration. When the ether groups interacted through hydrogen bonding with the OH groups of DGEBA, the electron density of the ether oxygen atoms decreased, resulting in a signal shift to lower wavenumber. The main peak of the ether shifted from 1110 to 1105 cm^−1^ and became sharp when the DGEBA concentration was increased. Moreover, a shoulder signal arose at 1074 cm^−1^ upon increasing the DGEBA concentration; this signal represented the ether groups of the PPO segment hydrogen bonded with the OH groups of the DGEBA resin.

### 3.2. Thermal Analyses 

**Figure 3:**
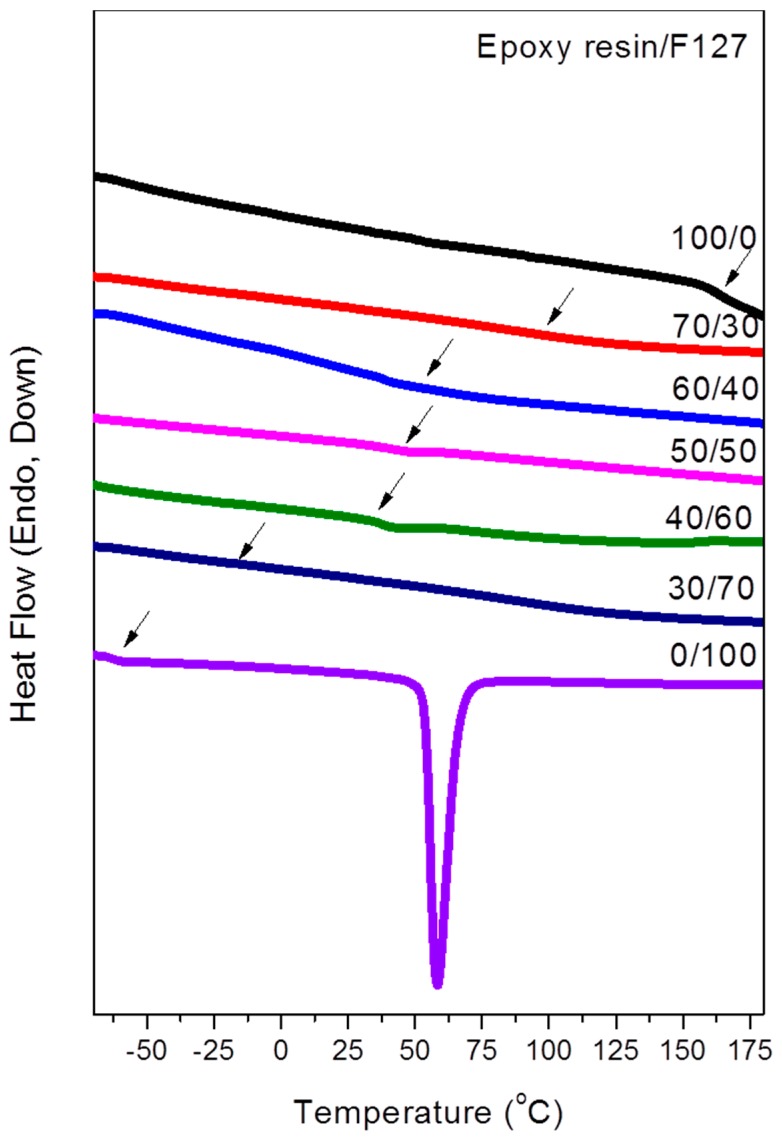
Differential scanning calorimetry (DSC) thermograms of epoxy resin/F127 blends of various compositions (second run; heating rate: 20 °C/min).

**Figure 4:**
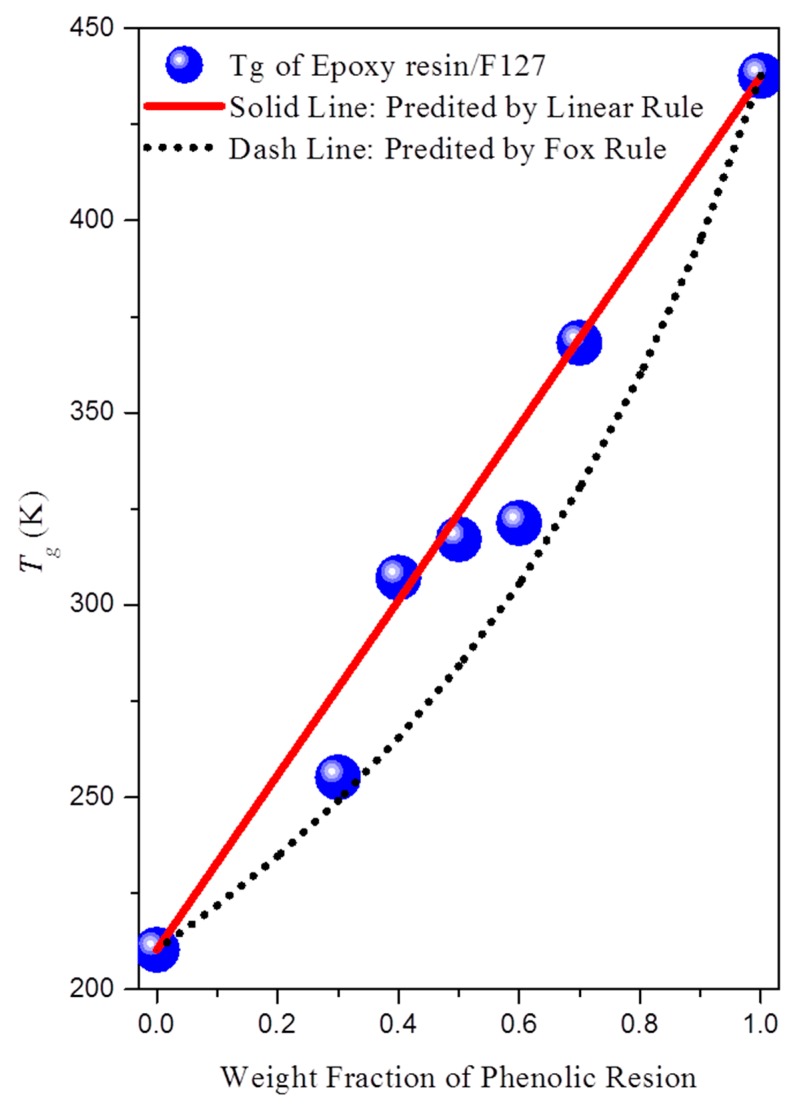
Glass transition temperatures (*T*_g_) of epoxy resin/F127 blends at various compositions.

The samples were heated to form bulk thermoset materials of epoxy/F127 blends. DSC has been used extensively to investigate miscibility behavior in polymer blends. The glass transition temperatures (*T*_g_) of the pure polymers of the PEO and PPO block segments were both approximately −62 °C; that of the epoxy resin used in this study was approximately 165 °C. Figure 3 presents the conventional second-run DSC thermograms of epoxy/F127 blends of various compositions after curing, obtained at a heating rate of 20 °C/min. The melting temperatures of pure F127 were mixed into one peak near 65 °C. The disappearance in melting temperature can be caused by morphological effects as well as thermodynamic reasons (e.g., hydrogen bonding interaction with epoxy resins); thus, we observed that the melting temperatures of the PEO and PPO blocks disappeared upon increasing the concentration of epoxy resin. The PEO and PPO blocks have no melting point after curing blend with Epoxy resin, because there is not enough area to crystallize after reaction-induced microphase separation. Table 1 summarizes the thermal properties of the epoxy resin/F127 blends, based on the DSC analyses. Because the values of *T*_g_ of PEO and PPO are very close, we could not determine the miscibility between PEO and PPO using one or two values of *T*_g_. The one value of *T*_g_ of PEO shifted to higher values upon increasing the content of epoxy resin from 30 to 70 wt %. This value of *T*_g_ presumably arose from the epoxy/PEO phase, because the strength of inter-association between the epoxy OH groups and the PEO ether groups was greater than the strength of inter-association between the epoxy OH groups and the PPO ether groups. Figure 4 reveals that a linear rule could be used to predict the single values of *T*_g_ for the epoxy resin/F127 blend; these values were higher than those predicted using the Fox rule. Next, we used TEM, SAXS, AFM, and DMA analyses to investigate the morphological transformations, self-organized structures, and mechanical properties of these immiscible epoxy/F127 blends.

### 3.3. Phase Behavior of Epoxy Resin/F127 Blends

**Figure 5:**
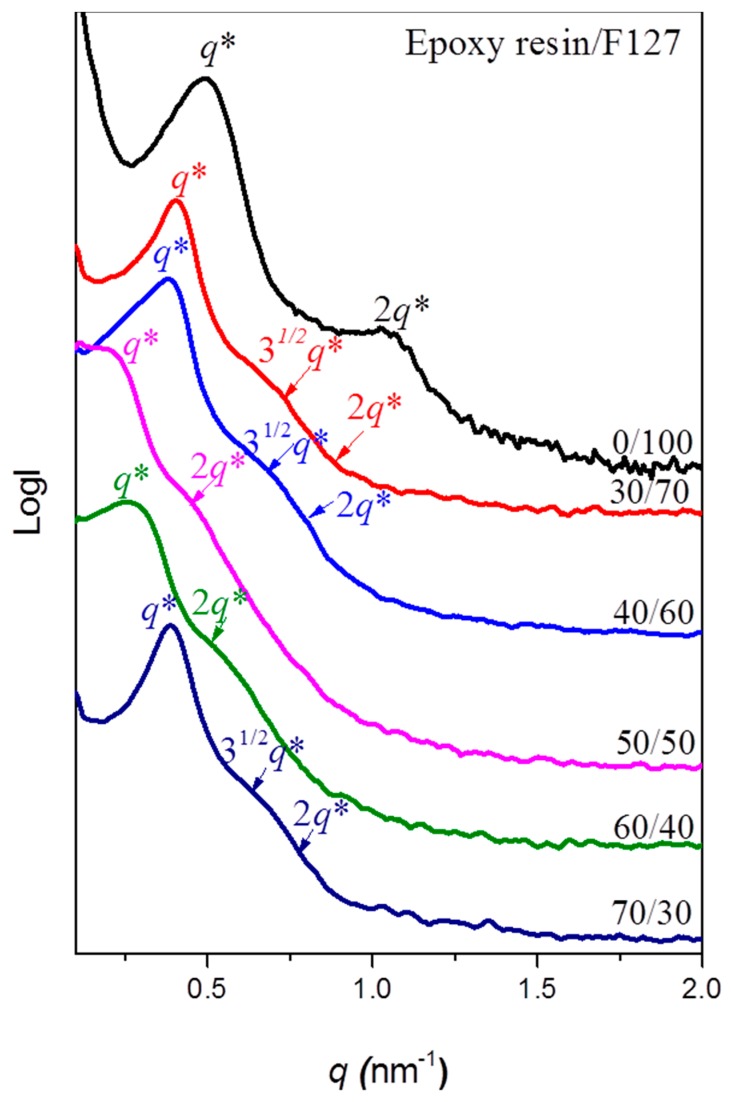
Small-angle X-ray scattering (SAXS) patterns of epoxy/F127 blends cured at weight fractions of F127 of 30, 40, 50, 60 and 70 wt %.

**Figure 6:**
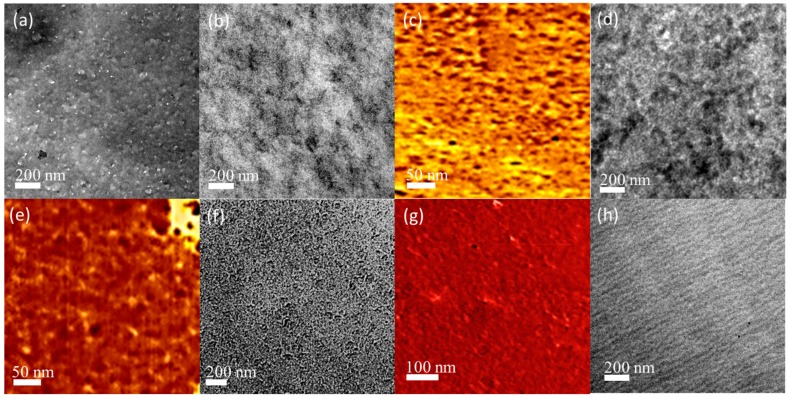
(**a**) Transmission electron microscopy (TEM) images of epoxy/F127 = 70/30; (**b**) TEM images of epoxy/F127 = 60/40; (**c**) AFM images of epoxy/F127 = 60/40; (**d**) TEM images of epoxy/F127 = 50/50; (**e**) AFM images of epoxy/F127 = 50/50; (**f**) TEM images of epoxy/F127 = 40/60; (**g**) AFM images of Epoxy/F127 = 40/60; (**h**) TEM images of epoxy/F127 = 30/70.

We recorded SAXS profiles (Figure 5) of the epoxy resins blended with the F127 triblock copolymer at room temperature to confirm the self-organized morphologies. An obvious and sharp primary scattering peak appeared for these blend systems; Table 1 summarizes the corresponding *d*-spacings, based on the Bragg equation. The profile of the pure triblock copolymer F127 revealed lamellar character, as determined by the specific ratio of q* and 2q* [32]. We observed some peaks having a ratio of q*, 3^1/2^q*, and 2q* at epoxy resin contents of 30 and 40 wt %, corresponding to the long period of hexagonally packed cylinders. Further increasing the epoxy resin content led to a phase transfer with different peaks in a ratio of q* and 2q* and an increased *d*-spacing. Furthermore, the profile of epoxy/F127 = 70/30 featured peaks in the ratio q*, 3^1/2^q*, and 2q*, with a decreased *d*-spacing, due to incomplete disordering of the hexagonal cylinders in this blend system. Figure 6 illustrates the different types of self-assembled morphologies observed in TEM and AFM images. Disordered micelle structures (the rich white parts provided by F127, a few dark parts provided by the epoxy resin) were evident at epoxy resin contents of 30 and 40 wt % (Figure 6a,b). Similarly, the AFM phase image in Figure 6c reveals two components at a 40 wt % epoxy resin content: a white region provided by F127 and a dark region provided by the epoxy matrix. The phase transition of the structures improved after increasing the epoxy content to 50 and 60 wt % (Figure 6d,f), consistent with the SAXS data (Figure 5). These images suggested that phase inversion had occurred, with only a few white parts provided by F127 and a rich excess of dark parts provided by the epoxy resin, similar to the AFM phase images in Figure 6e,g. For the epoxy resin/F127 = 70/30, we observed individual disordered cylindrical nanophases, which resulted from the PEO domains being imbedded in the continuous epoxy matrix, with an average diameter of approximately 10–15 nm. The formation of nanostructures in these thermosets templated by the amphiphilic block copolymers presumably occurred through so-called reaction-induced microphase separation (MIPS).

### 3.4. Mechanical Properties of Epoxy Resin

**Figure 7:**
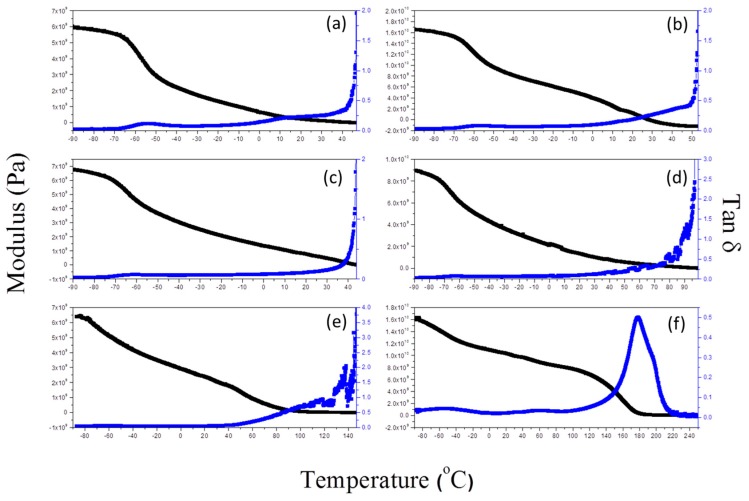
Dynamic mechanical analysis (DMA) of epoxy/F127 blends cured at weight fractions of (**a**) 30; (**b**) 40; (**c**) 50; (**d**) 60; (**e**) 70 and (**f**) 100 wt %.

Figure 7 displays the mechanical properties of the epoxy resin/F127 blends, determined through DMA, as well as their storage moduli and tan δ–temperature curves. The value of *T*_g_ of the epoxy resin was approximately 180 °C (Figure 7f); the value of *T*_g_ of the PPO segment, which we could not determine in the DSC analyses, was in the approximate range from −55 to −60 °C and independent of the epoxy resin content. In addition, the samples soften increased temperature larger than *T*_g_ (epoxy resin/F127 blend from DSC, Table 1). Bates *et al.* investigated the impact of different nanostructures on the mechanical properties of thermoset blends with block copolymers [14]. The toughening effect is significantly dependent on the second phase’s shape and size, and the interactions of the second phase within the thermoset matrix. They found from a study of the toughening mechanisms of wormlike micelle–modified epoxy resins that wormlike micelles have a high aspect ratio and, at an optimal length scale, might result in greater toughness than that of other nanostrucutures [14]. Figure 7 displays a modulus of this blend system, suggesting significantly enhanced toughness relative to F127 block copolymer. The modulus deceased upon increasing the F127 concentration and was less than 10^9^ Pa when the epoxy resin content was less than 50 wt % at 25 °C. Table 1 also summarizes the modulus data. As a result, we believe that the formation of wormlike micelles of flexible block copolymer segments in the surrounding epoxy resin can be a promising approach toward improving its toughness.

## 4. Conclusions

We have prepared flexible epoxy resins through a RIMPS mechanism upon blending with the triblock copolymer F127. We have used FTIR spectroscopy, DSC, SAXS, TEM, AFM, and DMA to investigate the miscibility, self-assembly behavior, hydrogen bonding interactions, and mechanical properties of the resulting epoxy resin/F127 triblock copolymer blends. FTIR spectroscopy provided evidence that the ether groups of the PEO and PPO segments functioned as hydrogen bond acceptors for the OH groups of the DGEBA epoxy resin. The SAXS data and TEM and AFM images revealed the presence of different microphase-separated structures within the epoxy resin/F127 blends at various compositions, each mediated through hydrogen bonding interactions. DSC and DMA provided thermal and mechanical data for these blends. We observed evidence for the formation of flexible epoxy resins as a result of wormlike micelles, with a high aspect ratio and an optimal length scale, of F127 acting as the second phase, resulting in greater toughness. The modulus data provided evidence for the wormlike micelles forming, through RIMPS, flexible thermosetting materials from the epoxy resin/triblock copolymer F127 blends.

## Figures and Tables

**Table 1:** Textural properties.

Sample	D (nm) ^1^	Modulus (Pa) at 25 °C	*T*_g_ (°C)	Epoxy Resin/F127
100%	-	1 × 10^10^	164.52	100/0
70%	16.1	2.23 × 10^9^	98.23	70/30
60%	24.5	1.16 × 10^9^	48.22	60/40
50%	28.5	6.07 × 10^8^	43.98	50/50
40%	16.6	4.59 × 10^8^	33.96	40/60
30%	15.5	1.75 × 10^8^	−17.97	30/70
F127	12.8	-	−62.84	0/100

^1^ The *d*-spacing values were calculated from the first small-angle X-ray scattering (SAXS) peak by the formula *d* = 2π/q*.
